# TaxMan: a taxonomic database manager

**DOI:** 10.1186/1471-2105-7-536

**Published:** 2006-12-18

**Authors:** Martin Jones, Mark Blaxter

**Affiliations:** 1 Institute of Evolutionary Biology, King's Buildings, Ashworth Laboratories, West Mains Road, Edinburgh EH9 3JT, UK

## Abstract

**Background:**

Phylogenetic analysis of large, multiple-gene datasets, assembled from public sequence databases, is rapidly becoming a popular way to approach difficult phylogenetic problems. Supermatrices (concatenated multiple sequence alignments of multiple genes) can yield more phylogenetic signal than individual genes. However, manually assembling such datasets for a large taxonomic group is time-consuming and error-prone. Additionally, sequence curation, alignment and assessment of the results of phylogenetic analysis are made particularly difficult by the potential for a given gene in a given species to be unrepresented, or to be represented by multiple or partial sequences. We have developed a software package, TaxMan, that largely automates the processes of sequence acquisition, consensus building, alignment and taxon selection to facilitate this type of phylogenetic study.

**Results:**

TaxMan uses freely available tools to allow rapid assembly, storage and analysis of large, aligned DNA and protein sequence datasets for user-defined sets of species and genes. The user provides GenBank format files and a list of gene names and synonyms for the loci to analyse. Sequences are extracted from the GenBank files on the basis of annotation and sequence similarity. Consensus sequences are built automatically. Alignment is carried out (where possible, at the protein level) and aligned sequences are stored in a database. TaxMan can automatically determine the best subset of taxa to examine phylogeny at a given taxonomic level. By using the stored aligned sequences, large concatenated multiple sequence alignments can be generated rapidly for a subset and output in analysis-ready file formats. Trees resulting from phylogenetic analysis can be stored and compared with a reference taxonomy.

**Conclusion:**

TaxMan allows rapid automated assembly of a multigene datasets of aligned sequences for large taxonomic groups. By extracting sequences on the basis of both annotation and BLAST similarity, it ensures that all available sequence data can be brought to bear on a phylogenetic problem, but remains fast enough to cope with many thousands of records. By automatically assisting in the selection of the best subset of taxa to address a particular phylogenetic problem, TaxMan greatly speeds up the process of generating multiple sequence alignments for phylogenetic analysis. Our results indicate that an automated phylogenetic workbench can be a useful tool when correctly guided by user knowledge.

## Background

Recently, there has been much interest in the use of large, concatenated multiple sequence alignments ('supermatrices') for phylogenetic analysis [1,2]. Such datasets have been shown to be useful in resolving difficult phylogenetic questions with a high degree of confidence. By combining the phylogenetic signal from multiple genes, clades can be recovered that are not recovered under analysis of any of the individual genes. Additionally, genes evolving at different rates may offer resolution at different phylogenetic levels.

Large-scale phylogenetic analyses of the type described above place a heavy burden of sequence acquisition, dataset assembly and dataset storage on the researcher. Sequences corresponding to the genes of interest must be obtained from public databases and orthology assigned. Where multiple sequences are available for a given gene in a species (as is often the case with EST datasets, for example) a consensus sequence must be derived. The sequences for each gene must then be aligned before being added to a concatenated alignment file, which may also contain commands necessary to partition the data. The forest of trees resulting from subsequent phylogenetic analysis must be associated with the relevant datasets, analysis parameters and confidence metrics. With these tasks in mind we have developed an integrated solution, TaxMan. TaxMan reduces this curatorial burden by automatically assembling and storing large aligned sequence datasets, and also storing metadata and trees resulting from phylogenetic analysis. Because of the high level of automation offered by TaxMan, datasets can be rebuilt rapidly to include new sequence data.

## Implementation

**Figure 1:**
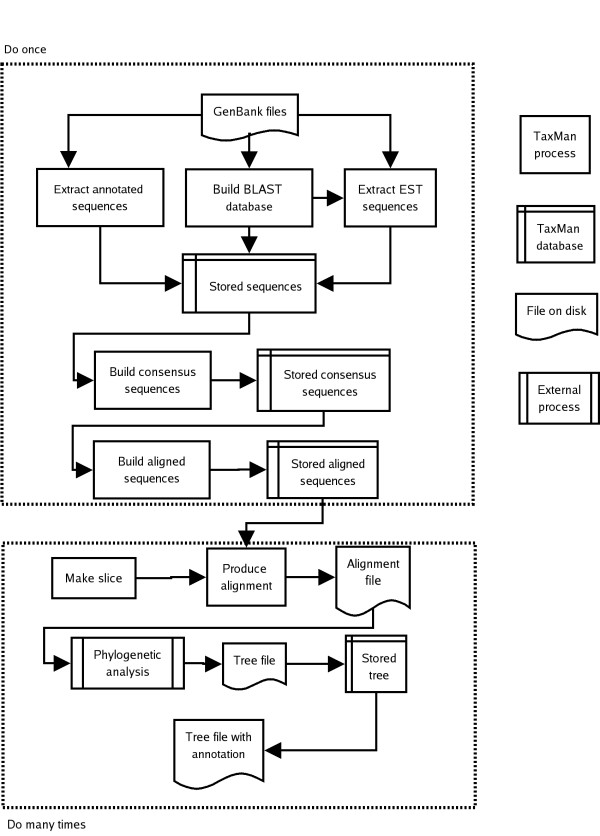
Diagram of the TaxMan workflow showing the steps involved in multigene phylogenetic analysis. The upper dotted box shows the stages that are carried only once, to build the database – sequence acquisition, consensus building and alignment. The lower dotted box shows the stages that are typically carried out multiple times – definition of a subset of genes and taxa, production of an alignment file, subjecting the alignment to phylogenetic analysis and storing the resulting tree.

TaxMan is written in Perl [see Additional file 1] and makes extensive use of modules from the BioPerl project [3]. PostgreSQL, a relational database management system (RDBMS), is used to store data. TaxMan makes use of many freely available bioinformatics tools; see the user guide [see Additional file 2] for a complete list and details of how to obtain them. The steps required to build a database of aligned genes are threefold; sequence acquisition, consensus derivation and alignment. Figure 1 gives an overview of the TaxMan workflow.

### Database construction

TaxMan extracts DNA sequence data from GenBank format [4] files on the basis of either annotation or sequence similarity to a database of identified genes. The NCBI Entrez service [5] allows a user to quickly select sequences from a given taxonomic group for download, and to filter out large records (e.g. chromosomes) that may cause problems. Curated genome-scale datasets of coding sequences can be included by downloading them from the relevant genome database. The user must supply a pre-defined list the genes for which they wish to extract records, and, for each, a list of synonyms – for example, cytochrome oxidase subunit 1 is variously annotated as 'COX1', 'CO1', 'COI', etc. When extracting sequence data based on annotation, TaxMan reads each GenBank record from a file and examines all features tagged as 'CDS' or 'rRNA'. Features with annotation matching that of one of the genes of interest are stored. To extract records on the basis of similarity, the user must supply a datasets of sequences known to be members of the target gene set. This dataset can be generated automatically from a GenBank format file. TaxMan uses BLAST [6] to identify sequences with significant similarity to known representatives of the gene set. Sequences extracted on the basis of annotation are referred to as 'annotated sequences'; those extracted on the basis of sequence similarity are referred to as 'screened sequences'. Screened sequences are normally ESTs, but may also be full length CDSs that were not annotated, or genomic fragments. By extracting and storing only sequence data that are of interest, TaxMan can cope with extremely large input datasets (e.g. all 1.5 million GenBank CoreNucleotide records for the phylum Arthropoda).

TaxMan assumes that all sequences with a given user-assigned gene name are orthologues, and a single definitive sequence is derived for each gene in each species. Users must take care to specify synonyms for genes in which they can be confident of orthology. A simple rule-based approach is used, whereby sequences from fully sequenced genomes are preferred over "annotated" sequences, which are preferred over "screened" sequences. Where multiple sequences of the same type are available, phrap [7] is used to generate a consensus sequence which is stored in the database. Annotation in GenBank may contain errors, and these measures are designed to minimise their effect.

For each species, metadata derived from the GenBank accession, including the unique 'taxid' identifying the species of origin, are also stored. All *a priori *taxonomic information stored in TaxMan is extracted from the NCBI taxonomic database [8]. Although the NCBI taxonomy is not an authoritative source, it is the framework within which sequences are stored, and is the only complete source of information regarding the relationships between the taxa that are represented in GenBank. Support for alternative taxonomies is a possible future feature of TaxMan (see limitations and extensions below).

For each gene, consensus sequences from all taxa are aligned using POA [9]. POA performs global multiple sequence alignment which, in preliminary testing, was found to cope well with partial sequences that are often found in datasets assembled by TaxMan. Other alignment programs can be used with relatively straightforward changes to the program code. The alignment process is different for protein-coding and rRNA genes. For protein-coding genes, all "annotated" sequences are translated to protein based on the GenBank-annotated start and stop codons and the protein sequences aligned. The DNA sequences are then back-aligned on the protein alignment using the transeq program from the EMBOSS package [10]. Finally, the "screened" sequences are added to the DNA alignment. The individual aligned protein sequences and aligned DNA sequences are stored in the database. For RNA genes, all sequences are aligned simultaneously and the aligned sequences stored as DNA. Importantly, TaxMan does not carry out any type of alignment quality control; alignments should be checked either manually or using an objective score function such as norMD [11]. Manual inspection of alignments will also uncover any erroneous sequences introduced by incorrect GenBank annotations.

### Output of datasets for phylogenetic analysis

Once a set of aligned sequences has been generated and stored, TaxMan can produce concatenated multiple sequence alignment files containing subsets of genes and taxa (called 'slices') in a partitioned format suitable for phylogenetic analysis. Slices can be specified manually by the user, or TaxMan can select slices automatically by including a chosen number of representatives from each group at a given taxonomic level, determined using the NCBI taxonomy. Specifying a slice in this way allows an alignment to be produced that has wide taxonomic sampling while keeping the total number of taxa computationally tractable. TaxMan produces alignments in Nexus, Phylip and FASTA file formats, suitable for analysis with a range of phylogenetic software packages. Alignments output from TaxMan in Nexus format contain character set commands partitioning the alignment by gene and by codon position.

### Storing the results of phylogenetic analysis

TaxMan can also store trees resulting from phylogenetic analysis. The structure of a tree is stored in the form of individual nodes, which allows storage of branch lengths and support values, along with links to species data for the terminal nodes. By storing multiple data types together in a database, GenBank records, consensus sequences, aligned sequences, alignments and trees can be collected in one place and much record-keeping taken care of automatically.

## Results

TaxMan was used to assemble a dataset for seventeen loci, including mitochondrial protein-coding (all 13 animal mitochondrial protein-coding genes) and ribosomal RNA genes (12S and 16S), and nuclear ribosomal RNA genes (SSU, LSU), from the subphylum Chelicerata. Approximately 12,000 annotated CoreNucleotide records and 74,000 EST records were downloaded from the NCBI GenBank database using Entrez [5](15/10/2005). TaxMan extracted 5,410 annotated sequences and 8,807 screened sequences using a chelicerate mitochondrial genome BLAST database. 1,204 species were included, representing 3 classes, 17 orders and 142 families. 2,398 consensus sequences were generated (1,992 from annotated sequences, 136 from EST sequences, 270 from fully sequenced mitochondrial genomes) and aligned. At this point, subsets of genes and taxa were specified and concatenated alignment files generated for phylogenetic analysis. For example, a slice was automatically specified that contained all genes and 3 representative species per order, selecting taxa with the most sequence available. The alignment file resulting from this slice was analysed to investigate the relationships between the chelicerate orders [12]. The process took approximately two hours (not including phylogenetic analysis) on a Linux server (Intel Xeon 3.4 GHz processor and 2 GB of RAM).

## Discussion

We envisage the target users of TaxMan as researchers with experience of phylogenetics who are interested in assembling and curating a large set of aligned sequences and resulting trees for a taxonomic group for multiple-gene phylogeny. Using TaxMan, the user can assemble a large dataset of aligned DNA and protein sequences for multiple genes much more quickly and more completely than could be achieved manually. It can cope with very large input datasets and also facilitates selection of taxa for analysis and curation of phylogenetic results. Other phylogenetics software packages do not fulfil the role of TaxMan. Some packages carry out a single step in the phylogenetic process (for example, HAL [13] attempts to define orthologues in a sequence dataset using sequence similarity, and the Phylota [14] project contains applications for database clustering and subset definition) but lack integration. ARB [15] is a computing environment for phylogenetic analysis of DNA sequences, originally developed for RNA genes, but it cannot cope with multiple genes or construct consensus sequences. TreeBlaster [16] gathers sequence data based on BLAST similarity and uses phylogenetic software tools to construct a tree, but cannot use multiple genes, select sets of sequences for analysis, build consensus sequences or store phylogenetic results. The Arthropodan Mitochondrial Genomes Accessible database (AmiGA [17]) produces concatenated alignments for multiple genes, but requires that slices are specified prior to alignment, resulting in much greater processing times.

TaxMan is limited by design in two main ways; firstly, it is incapable of storing multiple reference taxonomies, instead taking the NCBI taxonomy as a base. Secondly, it assumes that a single consensus sequence is to be generated for each locus in each species, making it unsuitable for sampling from gene families, or from populations from within species (as found, for example, in DNA barcoding studies). Planned future directions for TaxMan include support for multiple taxonomies and for multiple sequences per locus. A mechanism allowing users to easily exclude sequences that have been incorrectly identified would be of benefit – currently, sequences must be deleted manually from the database. Routines for automatic orthology assignment, a significant problem in its own right, could also be included. The ability of TaxMan to store multiple phylogenetic trees for a given set of terminal taxa opens up additional possibilities for automated tree comparison tools.

## Conclusion

There are many scenarios in which researchers might want to assemble a dataset of aligned DNA or protein sequences with a view to phylogenetic analysis. Current approaches require assembling such datasets manually or, at best, using software tools that lack integration. TaxMan greatly facilitates the various steps required for a multigene phylogenetic study by extracting sequences from public databases, building and aligning consensus sequences, choosing sets of data for analysis and storing the results of analysis. By using a relational database to store data, along with existing bioinformatics tools and a Perl framework, TaxMan allows large datasets to be assembled extremely rapidly and with full data provenance, allowing the user to concentrate on the analysis.

## Availability and requirements

Project name: TaxMan

Project home page: 

Operating system: Linux

Programming language: Perl (5.8.6)

Other requirements: BioPerl (1.4), CPAN, PostgreSQL (8.0.8), BLAST (2.2.13), EMBOSS (3.0.0), phrap (0.990329), POA

License: GNU GPL

Restrictions: None

## Authors' contributions

MJ wrote and documented the software and wrote the manuscript. MB assisted with design and testing of the software and writing of the manuscript, and supervised the project. Both authors read and approved the manuscript.

## Supplementary Material

Additional file 1 TaxMan. The TaxMan software package Click here for file

Additional file 2 TaxMan user guide. User guide for the TaxMan software package Click here for file
