# Supplementary material for: TaxMan: a taxonomic database manager
Source: BMC Bioinformatics. 2006 Dec 18;7:536. doi: 10.1186/1471-2105-7-536 (PMC1766369; doi:10.1186/1471-2105-7-536)
Supplement: Additional file 1 — TaxMan. The TaxMan software package [file 1471-2105-7-536-S1.gz › taxman_1.1/user_guide.pdf]

TaxMan

Taxonomy Database Manager

User guide
Version 0.11

Martin Jones
martin.jones@ed.ac.uk

Table of Contents

About this user guide....3
Glossary....4
1 Introduction to TaxMan....6
1.1 What TaxMan does....6
1.2 What TaxMan doesn't do....6
2 Before you run TaxMan....6
2.1 Dependencies....6
2.1.1 Perl....7
2.1.1.1 The Perl interpreter....7
2.1.1.2 BioPerl....7
2.1.1.3 Modules....7
2.1.2 External programs....8
2.1.2.1 BLAST....8
2.1.2.2 phrap....8
2.1.2.3 tranalign from EMBOSS....8
2.1.2.4 poa....8
2.1.3 Database....9
2.2 Running the test script....9
2.3 Preparing files for processing with TaxMan....9
2.3.1 Config file....9
2.3.2 GenBank sequence files....10
2.3.3 NCBI taxonomy files....11
2.4 Running TaxMan....11
3 Setting up the database....11
3.1 Creating a new database....11
3.2 Database structure....11
3.3 Inserting gene information....12
4 Adding sequences....12
4.1 Adding annotated sequences....12
4.1.1 Checking for common gene names in an annotated sequence file....12
4.1.2 Inserting annotated sequences from a GenBank file....13
4.2 Adding EST sequences....13
4.2.1 Building a BLAST database....13
4.2.1.1 Hand-pick sequences....14
4.2.1.2 Pick a set of species, and use the same GenBank file you used to add annotated sequences....14
4.2.2 Reading a GenBank file....15
4.3 Adding species to the database....15
5 Building and aligning consensus sequences....15
5.1 Making consensus sequences....16
5.1.1 Making gene counts....16
5.1.2 Building consensus sequences....16
5.2 Aligning sequences....17
6 Working with slices....17
6.1 Looking at the distribution of data....17
6.2 Automatically creating slices....18

6.2.1 Creating default slices....18
6.2.2 Creating slices based on taxonomic level....19
6.3 Manually creating slices....19
7 Producing an alignment....19
7.1 Alignment names and formats....19
7.2 DNA alignments....20
7.2.1 Alignments with taxids....20
7.2.2 Alignments with species names....20
7.3 Protein alignments....21
7.3.1 Alignments with taxids....21
7.3.2 Alignments with species names....21
8 Working with trees....21
8.1 Storing trees....21
8.1.1 Storing the NCBI taxonomy....21
8.1.2 Storing a newick/nexus tree....22
8.2 Outputting trees....22
8.2.1 Types of annotation....22
8.2.2 Producing a tree....22
Alphabetic Index....23
Workflow diagram....25
Database diagram....26
Genetic Codes....27

About this user guide

The user guide is intended to inform the user about how to use TaxMan and about some of the biological thinking behind TaxMan's design. However, it is no substitute for basic knowledge of biology, bioinformatics and phylogenetics. TaxMan will not stop you if you try to do something that does not make biological sense. Do not be tempted to treat it as a black box!

Several formatting conventions are used throughout the user guide:

`Commands that are meant to be typed, text within files and TaxMan output is written in this style`

Names of programs, packages, files and formats are written in this style. Items in this style usually have an entry in the glossary

<u>References to web sites are written in this style – mostly telling you where to download software</u>

Citations of papers describing software tools are written in this style

Glossary

A brief description of words that are in **bold** in the text.

Words used by TaxMan

**annotated sequence**	A sequence that has been identified by TaxMan on the basis of annotation in a GenBank file
**EST**	Expressed Sequence Tag; a type of sequence that lacks annotation. TaxMan identifies EST sequences on the basis of BLAST similarity
**taxid**	A number used in the NCBI to represent a taxonomic group
**tree_id**	A number that uniquely identifies a stored tree
**slice_id**	A number that uniquely identifies a slice

File formats

**GenBank**	A sophisticated file format for storing sequences and metadata
**fasta**	A simple file format for storing sequences
**csv**	Comma Separated Values; a file format for storing tables
**phylip**	A file format for storing aligned sequences (and a set of phylogenetics programs)
**nexus**	A sophisticated file format for storing aligned sequences, that can include character sets and other commands

Files / Directories

**BLOSUM80.mat**	A text file describing the patterns of change of amino acids
**taxman.config**	A text file containing configuration information for TaxMan
**postgres.log**	A file containing warning messages from PostgreSQL
**blast_database_files**	A directory where TaxMan stores BLAST databases
**allpep**	BLAST database holding protein-coding genes
**allrna**	BLAST database holding rRNA genes
**taxdump.tar.gz**	A compressed file containing the NCBI taxonomy

Software

**TaxMan**	The software you are reading about; short for Taxonomic database Manager
**Perl**	A high-level programming language, popular in bioinformatics
**PostgreSQL**	An open-source relational database management system
**BioPerl**	A collection of Perl modules to carry out common bioinformatics tasks
**Linux**	An open-source operating system widely used in bioinformatics
**phrap**	A program used to cluster similar sequences into contigs
**blastall**	The executable for carrying out BLAST searches
**formatdb**	The executable for formatting BLAST databases
**EMBOSS**	A package of sequence analysis software tools
**tranalign**	A tool from the EMBOSS package for aligning DNA sequence based on a protein alignment
**POA**	A multiple sequence alignment program
**BLAST**	Basic Local Alignment Search Tool; used to identify similarity between sequences
**blastclus**	A program that can identify redundant sequences

1 Introduction to TaxMan

1.1 What TaxMan does

TaxMan (Taxonomy database Manager) is a Linux program for assembling and investigating large phylogenetic datasets, containing multiple genes for large taxonomic groups. It consists of a database used to store sequence and other data, and a number of Perl modules, controlled by a menu-driven user interface. TaxMan can:

- Rapidly search within GenBank files for sequences of interest
- Search EST datasets to extract sequences of interest
- Automatically construct consensus sequences
- Automatically carry out alignments and store aligned sequences
- Produce large concatenated alignment of multiple genes and set up character partitions
- Store and retrieve trees resulting from phylogenetic analysis

1.2 What TaxMan doesn't do

In order to achieve a high level of automation, TaxMan makes several assumptions about the type of dataset you are working with. TaxMan does not:

- Deal with multi-copy genes
- Automatically cluster sequences or try to assign orthology
- Carry out any tree-building algorithms

2 Before you run TaxMan

TaxMan relies on a number of external components to run smoothly. These include **Perl** modules (and the **Perl** interpreter), external standalone programs and the **PostgreSQL** relational database management system. This chapter gives details of each of the dependencies, where they are used in **TaxMan** and how they can be obtained. It also describes the test script that is part of the **TaxMan** distribution. This script will test all of the various dependencies required by **TaxMan** and inform you of any that are missing. If you're missing **Perl**, of course, you won't be able to run the test script until you've installed it.

2.1 Dependencies

The software required by **TaxMan** falls into three categories: **Perl** (comprising the **Perl** interpreter, **BioPerl** and additional modules); external bioinformatics programs (such as **BLAST**) and **PostgreSQL**, a relational database management system (RDBMS).

2.1.1 Perl

Perl is an interpreted programming language that is particularly suited to bioinformatics programs for a number of reasons. It has extremely advanced string manipulation functions, which work quite happily on strings of sequence data. It is also relatively quick to learn and can be used to write useful applications in a very short period of time. Perhaps most importantly, there exist a wide range of **Perl** modules, libraries containing code that carries out common tasks. For bioinformatics there is the **BioPerl** project, a collection of modules that carry out common tasks such as reading sequence files and carrying out similarity searches. The **Perl** dependencies of **TaxMan** are threefold: the **Perl** interpreter, the **BioPerl** project and a number of additional **Perl** Modules.

2.1.1.1 The Perl interpreter

The **Perl** interpreter is usually present on most **Linux** distributions; running the command

```
which perl
```

from a terminal window should return a line giving the location of the **perl** executable, provided it is in your path. If **Perl** is not installed it can be obtained from http://www.cpan.org/.

2.1.1.2 BioPerl

BioPerl is not usually installed on **Linux** distributions and will normally have to be installed separately. You can find out if it is installed by running

```
perl -MBio::Perl -e '1'
```

from a terminal window; if it doesn't return an error then **BioPerl** is installed. If it is not installed it can be obtained by following instructions from http://bioperl.org/wiki/Installing_BioPerl.

2.1.1.3 Modules

The modules that are required by **TaxMan** that are not part of the standard **Perl** installation are as follows:

DBI
DBD::Pg
Math::Combinatorics
Term::ANSIColor

Modules that are not installed can be obtained from http://www.cpan.org/modules/index.html; instructions on how to install modules can be found at http://www.cpan.org/modules/INSTALL.html. Most of the time, a command similar to

```
perl -MCPAN -e 'install DBI'
```

executed as root, will suffice.

2.1.2 External programs

TaxMan relies on a suite of external programs to carry out its functions. At the time of writing, these are all freely available on the web. For each program I will give the reference and the location at which it can be downloaded, and describe its role in TaxMan.

2.1.2.1 BLAST

http://www.ncbi.nlm.nih.gov/BLAST/download.shtml

Altschul, S F, Madden, T L, Schäffer, A A, Zhang, J, Zhang, Z, Miller, W and Lipman, D J. 1997. Gapped BLAST and PSI-BLAST, a new generation of protein database search programs. Nucleic Acids Res. 25:3389-3402

BLAST is used to search a formatted database for sequences that show similarity to a query sequence. Within **TaxMan**, it is used to identify EST sequences that show significant similarity to a known gene, and thereby to putatively assign orthology. It is also used to select the best consensus sequence in the event that **phrap** produces multiple sequences during the consensus building process. There are two executables that are required, **blastall** (which carries out the search) and **formatdb** (which prepares a database for searching).

2.1.2.2 phrap

http://www.phrap.org/consed/consed.html#howToGet

Gordon, David. "Viewing and Editing Assembled Sequences Using Consed", in Current Protocols in Bioinformatics,A. D. Baxevanis and D. B. Davison, eds, New York: John Wiley & Co., 2004, 11.2.1-11.2.43.

phrap is used to produce a consensus sequence from multiple input sequences. Within **TaxMan**, it is used during the consensus building process where multiple sequences are found for a given gene in a given species. By using **phrap** to combine the information in multiple sequences, we can obtain a consensus sequence that is longer and more accurate than any of the individual input sequences.

2.1.2.3 tranalign from EMBOSS

http://emboss.sourceforge.net/

Rice,P. Longden,I. and Bleasby,A. 2000 EMBOSS: The European Molecular Biology Open Software Suite. Trends in Genetics 16:276-277

The **EMBOSS** package is a collection of programs for sequence analysis. The **tranalign** tool uses a protein multiple sequence alignment to align a collection of corresponding nucleotide sequences. In **TaxMan**, it is used during the alignment step to produce nucleotide sequence alignments of CDS sequences.

2.1.2.4 poa

http://www.bioinformatics.ucla.edu/poa/welcome.html

Lee, C, Grasso, C and Sharlow, M F. 2002. Multiple sequence alignment using partial order graphs. Bioinformatics. 18:452-464

POA (partial order alignment) is a program for carrying out multiple sequence alignment using partial order graphs. Two files are necessary; the **poa** executable and a matrix file, **BLOSUM80.mat**, which is distributed with **TaxMan**. **POA** is the alignment program of choice for **TaxMan** because it is fast (necessary when dealing with large datasets) and local (necessary when aligning partially overlapping sequences such as those derived from ESTs).

2.1.3 Database

TaxMan uses **PostgreSQL,** a relational database management system (RDBMS) to store various pieces of information; sequences, annotation, alignments, etc. A relational database is an ideal way to store large datasets as it allows very fast storing and retrieval of information. **PostgreSQL** is an open-source RDBMS which uses the Structured Query Language (SQL) standard. It can be obtained from http://www.postgresql.org/download/. To run **TaxMan**, you (the user on the machine you want to use) must be able to create and access **PostgreSQL** databases. The **createuser** admin tool (http://www.postgresql.org/docs/8.1/static/app-createuser.html) may be of use here. A diagram of the TaxMan database structure is included at the end of this document.

2.2 Running the test script

The **TaxMan** distribution includes a test script that will check all of the above – Perl modules, external programs and databasing – and report any errors. To run it, just enter the the **test_script** directory and run the **test_script** executable:

```
cd test_script
./test_script
```

The script will first check that you have the correct version of **BioPerl** and the **DBD::Pg** module before checking for additional perl scripts. Before testing database access, the test script will pause to allow you to quit any programs that might currently be using **PostgreSQL**. It will then go on to test various database functions and verify the presence of external programs. Check the output for for any errors. If you need to install any dependencies, refer to the above sections.

2.3 Preparing files for processing with TaxMan

2.3.1 Config file

TaxMan reads configuration information, each time the program is started, from the file **taxman.config**. The config file is divided into three sections, each of which starts with a `[header]` line. The first section simply holds the name of the current working database. It contains a single line of the form

```
database = name
```

The second section contains the information about the genes that are going to be stored. Each line gives the 'canonical name' of the gene (i.e. the name by which it will be referred to in **TaxMan**), a list of synonyms for the gene (i.e. names that may appear in GenBank records) and the genetic code used to translate the gene in the alignment step (this allows us to mix mitochondrial and nuclear genes in one database). Genetic code numbers are taken from the NCBI (http://130.14.29.110/Taxonomy/Utils/wprintgc.cgi?mode=c) and are given at the end of this document. In the case of RNA genes, the canonical name must contain the string RNA so that **TaxMan** can recognise them; the genetic code does not matter as it is never used. The format is:

```
canonical name = synonym 1,synonym 2,synonym 3,genetic_code
```

for example:

```
EF1A = EF-1alpha,EF-1a,EF1a,EF1-alpha,elongation factor 1 alpha,1
```

You must be careful not to include any extra whitespace between synonyms; separate them with commas only. It's fine to include spaces in the synonyms. The genetic code must always be the last item on a line and must be followed by a newline.

The third section contains the list of taxa to be included in the **BLAST** database. The format is:

```
blast_taxa = taxid1,taxid2,taxid3
```

eg

```
blast_taxa = 59655,6938,34612
```

where taxids are taken from the NCBI taxonomy database. This list allows the user to specify a restricted set of taxa to be included in the **BLAST** database of sequences which will be used to screen ESTs. Doing this means that the size of the database is kept down and hence the time required for **BLAST**s is lower. This option will not make sense until you have read section 4.2.1

2.3.2 GenBank sequence files

The inputs for **TaxMan**, as far as sequence data is concerned, are GenBank format text files. A GenBank file contains multiple records, each of which contains a sequence, various metadata associated with the sequence, and information about features on the sequence such as CDSs and protein translations. This format is very flexible; a single GenBank record can correspond to an EST sequence, a complete mRNA sequence or even an entire chromosome. See http://www.ncbi.nlm.nih.gov/Sitemap/samplerecord.html for a sample record along with an explanation of the various fields.

Since annotated sequences (used in **TaxMan** to refer to any sequence that is tagged as a gene and has a name) and EST sequences are treated differently by **TaxMan**, it is best to download them separately as two GenBank files. This is easily done via the NCIB Entrez Nucleotide website (http://www.ncbi.nlm.nih.gov/entrez/query.fcgi?db=Nucleotide) which allows the user to select only records from the CoreNucleotide, EST or GSS portions of the GenBank database. By choosing CoreNucleotide or EST, then selecting 'GenBank' in the Display box and 'file' in the Send to box, one can easily download all records of a particular type to a single GenBank file. GenBank format files should be saved in the TaxMan directory.

2.3.3 NCBI taxonomy files

There are two processes in **TaxMan** which require a current version of the NCBI taxonomy; assigning species to taxonomic groups (order, species, etc. - see section 4.3) and producing taxonomic information for a subset of taxa (see section 8.2.2). The NCBI makes available a file containing a dump of the taxonomic database (currently at ftp://ftp.ncbi.nih.gov/pub/taxonomy/taxdump.tar.gz) which must be downloaded at the same time as the GenBank records. Extract the files in the same directory as **TaxMan**:

```
tar zxvf taxdump.tar.gz
```

2.4 Running TaxMan

To run **TaxMan**, ensure that the current working directory is the **TaxMan** directory (named **taxman_x.xx**, where **x.xx** is the version number). Run the **TaxMan** executable thus:

```
./taxman
```

If you see a menu entitled top level menu, all is well.

3 Setting up the database

When preparing to assemble a dataset using **TaxMan**, the first step is to create a database and prepare tables to hold the data. Once created, a database persists from session to session, i.e. you can quit **TaxMan** then run it at a later time and the data will still be available. The name of the database to create (and, later, to connect to) is given in the configuration file as described in section 2.3.1. The easiest way to clear all information in a database is simply to go through the database creation procedure again – any existing database with the same name as the one you are trying to create will be deleted. You don't have to run any command to connect to an existing database; when you select an option that requires it, **TaxMan** will automatically connect and disconnect as necessary

3.1 Creating a new database

At the `top level menu`, select `option 1 (Database Step 1)` then at the `Database step 1 menu` select `option 2 (Create database)`. If the database you want to create (loaded from the **taxman.config** file) already exists, you'll be warned and asked if you want to continue. If you do, the existing database will be deleted and a new one created. Any messages that **PostgreSQL** produces will be written to the file **postgres.log**

3.2 Database structure

There are 6 types of objects that are stored in the database – genes, species, raw sequences, consensus sequences, aligned sequences and trees. Each of the types of objects has a table to itself. Additionally, there are extra tables: alignment, which holds metadata for a particular set of aligned sequences, and node, which holds information about the nodes that make up a tree. See the diagram at the end of this document for table details.

3.3 Inserting gene information

In order to extract sequences of interest from the GenBank input files, TaxMan relies on a list of gene names and synonyms, which is read from the **taxman.config** file (section 2.3.1). To insert this information into the database, at the `top level menu`, select `option 1 (Database Step 1)` then at the `Database step 1 menu` select `option 3 (Insert gene info)`. The output will tell you the canonical names of the genes that are being inserted. The information will be stored in the gene table. If you have a GenBank file of records for the taxonomic group of interest, you might want to use **TaxMan** to check them for gene names (section 4.1.1), then edit the **taxman.config** file based on the output. If you do this, you'll have to quit and restart **TaxMan** in order for the **taxman.config** file to be re-read before inserting gene names.

4 Adding sequences

There are two sequence types recognised by **TaxMan**, and they are treated by the program in different ways. A particular sequence is assigned to one type or the other on the basis of how it was recognised as a gene of interest. There are two ways in which a sequence can be recognised as a gene of interest; by annotation, or by sequence similarity. If a sequence is read from a GenBank record and the gene name is found to be identical to one of the synonyms in the gene table, it is recognised by annotation and is treated as an **annotated sequence**. If, on the other hand, a sequence is found to have significant similarity to a sequence in a BLAST database built from annotated sequences it is treated as an Expressed Sequence Tag (**EST**). **Annotated sequences** and **ESTs** are treated differently in the consensus-building step and the alignment step.

4.1 Adding annotated sequences

To add annotated sequences you need to supply **TaxMan** with a GenBank file containing some records, having first inserted some gene names and synonyms into the database. It is important to remember that sequences will only be added in this step if they are annotated with a name that's identical to one of the synonyms in the gene table. Specifically, the program looks through GenBank records for features that are tagged '`gene`' or '`product`' or '`rRNA`' and whose '`gene`' or '`product`' tag is identical to one of the synonyms in the gene table. This allows for records that contain multiple genes of interest (e.g. mitochondrial genomes).

The two options detailed here ('checking for gene names' and 'reading a GenBank file') carry out similar processes but give different outputs. The first reads a GenBank file looking for gene names that occur commonly and are not already included in the gene table. The second actually stores the sequences in the database. You can run either of these options as many times as necessary on multiple GenBank files. Unlike inserting gene information (section 3.3), reading a GenBank file does not replace existing sequences in the database, but adds to them.

4.1.1 Checking for common gene names in an annotated sequence file

When you select this option, **TaxMan** looks through the records in a GenBank file to find annotated sequences, but instead of actually storing the sequences it keeps a record of commonly occurring gene names that are not identical to one of the synonyms in the gene table. This can be useful in two ways: firstly, one can identify synonyms for genes that were not previously known; secondly, one can identify genes that are well-represented in the dataset (and are therefore a good target for phylogenetic analysis). If you use this option and identify genes that you want to add to the gene table, you'll need to quit **TaxMan**, edit the **taxman.config** file to include the new gene information, then restart TaxMan and insert the new gene information into the database (section 3.3).

At the `top level menu`, select `option 2 (Database Step 2)` then at the `Database step 2 menu` select `option 1 (Check for common names in an annotated sequence file)`. **TaxMan** will prompt you to give the filename of a GenBank file. You should be able to use tab completion and line history editing. After you give the filename, **TaxMan** will search through all the records and report on any commonly occurring gene names. This may take a while if the GenBank file contains a large number of records – TaxMan will display a count of how many records have been processed. Remember that gene names which are already in the gene table (i.e. sequences annotated with them would have been added) won't be reported. If after viewing the output, you want to add any names to the **taxman.config** file, remember not to include any extra whitespace.

4.1.2 Inserting annotated sequences from a GenBank file

This option looks through the records in a GenBank file to find annotated sequences of interest, and stores them in the database. The process that gets carried out behind-the-scenes is very similar to that used when checking for gene names, so if you've run the process above on a particular GenBank file, you'll have an idea of how long it's going to take. At the `top level menu`, select `option 2 (Database Step 2)` then at the `Database step 2 menu` select `option 2 (Insert some annotated sequences)`. At the prompt, enter the name of a GenBank file. **TaxMan** will keep a running count of how many records have been processed and display it. After all records have read, **TaxMan** will display the number of sequences that were added.

All sequences (specifically, all features in GenBank records) that were not added to the database (i.e. were not annotated as a gene of interest) are saved to a separate GenBank file with the filename **unmatched_from_genbankfile.gb**. This is to allow the user to treat these records in the same way as ESTs, by using this new file as the input for adding EST sequences (section 4.2). By treating these sequences in the same way as ESTs, one can make use of sequence data that was not extracted during the adding CDS sequence step due to lacking or incorrect annotation.

4.2 Adding EST sequences

Sequences that have been added on the basis of **BLAST** similarity to a database of known genes (as opposed to being added on the basis of annotation) are treated by **TaxMan** as EST sequences. To add sequences on the basis of **BLAST** similarity requires that the user first build a **BLAST** database of sequences of known genes, then use the database to screen a file of sequences.

4.2.1 Building a BLAST database

When building a **BLAST** database, there is a trade-off to be made between speed and coverage. A database that contains many sequences for a particular gene, covering a wide taxonomic range of taxa within the group of interest, will have a higher chance of identifying similar EST sequences than a smaller database, but will take longer to search. In practice, the best approach is to just include sequences from a small number of species that span as much of the taxonomic diversity of your chosen group as possible, for which you know sequence data is available for all the genes of interest.

To search ESTs, **TaxMan** assumes that there are two formatted **BLAST** databases, **allpep** and **allrna**, in the **blast_database_file** directory. They can be generated manually or automatically by **TaxMan**. When automatically building **BLAST** databases, **TaxMan** uses a process very similar to that used when adding annotated sequences (section 4.1.2). The program looks through records in a GenBank file to identify sequences that are annotated as the genes of interest; instead of storing them in the database, it writes them out to a **FASTA** format file, which is then formatted as a **BLAST** database. It can also use a 'whitelist' of taxa, read from the **taxman.config** file, to ignore records that don't come from a set group of species. By way of example, here are three possible strategies for generating a 'good' **BLAST** database.

4.2.1.1 Hand-pick sequences

If your group of interest contains several species with lots of sequence data (possibly with fully-sequenced genomes), and those species represent a large proportion of the taxonomic diversity of the group then you can manually download the sequences for the genes of interest in those species. Save the sequences as a GenBank file, then use that file as the input for the make a **BLAST** database menu option. From the top level menu, select `option 2 (Database Step 2)` then at the `Database step 2 menu` select `option 3 (Make a BLAST database)`. At the prompt, enter the name of a GenBank file. Assuming that all sequences are annotated as genes of interest, they will all be included in the **BLAST** databases. **TaxMan** will automatically format the databases correctly.

4.2.1.2 Pick a set of species, and use the same GenBank file you used to add annotated sequences

The user can choose a set of species (actually a set of species level **taxids**) and enter them in the **taxman.config** file on a blast_taxa line, thus:

```
blast_taxa = 59655,6938,34612
```

If a `blast_taxa` line exists in the config file, **TaxMan** will only look in records belonging to that species when making the **BLAST** databases. After editing **taxman.config** to include the line, restart **TaxMan**. From the top level menu, select `option 2 (Database Step 2)` then at the `Database step 2` menu select `option 3 (Make a BLAST database)`. At the prompt, enter the name of the GenBank file that was used to add annotated sequences. **TaxMan** will extract only the sequences that belong to a species in the list, and are annotated as a gene of interest, and put them in the **BLAST** database. This is a lot quicker than the **"Handpick sequences"** method. The disadvantage is that, if there are several records for a gene in one of the listed species, there will be multiple redundant sequences in the **BLAST** database. One way to tackle this problem is to use an external program (e.g. **blastclus**) to remove redundant entries from the **allpep** and **allrna** **FASTA** files, then reformat the databases using **formatdb**.

Just use the existing CDS file

If there are few particularly well-represented species in your dataset, or they do not cover a lot of the taxonomic diversity, neither of the two options above will be very effective. In this case, you can simply use the same GenBank file you used to add annotated sequences as the input to the make a blast database menu option, with no blast_taxa line in the **taxman.config** file. From the top level menu, select `option 2 (Database Step 2)` then at the `Database step 2` menu select `option 3 (Make a BLAST database)`. At the prompt, enter the name of the GenBank file. In this scenario, all of the sequences that were stored in the database by the insert CDS menu option (section 4.1.2) will also be added to the BLAST databases. This method can result in very large **BLAST** databases that will make adding EST sequences very slow. Again, using an external program to remove redundancy from the **FASTA** files and reformatting may be a useful option.

4.2.2 Reading a GenBank file

After you've prepared your **BLAST** database, a GenBank file containing EST sequences can be added. Each sequence (specifically, each feature in each record – usually '`source`') is used as the query sequence in a similarity search of the **BLAST** databases. Any sequences that have a significant hit (<E-10) to one of the sequences of interest in the **BLAST** database are stored in the sequence table, and labelled as EST sequences. This can be the slowest part of the TaxMan process, if you have a lot of EST sequences downloaded. To add a file of EST sequences, from the top level menu, select `option 2 (Database Step 2)` then at the `Database step 2` menu select `option 4 (Add EST sequences)`. At the prompt, enter the name of the GenBank file that contains the EST sequences. Remember that you can use the file of non-matching sequences that was generated in the add CDS stage (section 4.1.2). When prompted, enter the genetic code you want to use for the **BLAST** searches. **TaxMan** will carry out the **BLAST** searches, read the results and store any sequences with significant hits. Progress will be displayed on the command line.

4.3 Adding species to the database

One of the pieces of metadata that is stored for each sequence is the NCBI taxid of the species from which it was obtained. In order to relate the taxid to other useful pieces of data (binomial name, classification, etc.) **TaxMan** must insert records for the relevant species into the species table. Do not carry out this step until all GenBank records (whether annotated sequences or EST sequences) have been inserted: only species which are represented in the sequences table will have data stored in the database. Species information is read from the NCBI taxonomy (section 2.3.3). The NCBI taxonomy dump file must be obtained at the same time as the sequence records. Assuming you have obtained the **taxdump.tar.gz** file and extracted as described in section 2.3.3, select `option 2 (Database Step 2)` from the top level menu then at the `Database step 2` menu select `option 5 (Put species and orders in the species table)`. **TaxMan** will parse the NCBI taxonomy files and store data for each species that is represented in the sequences table.

5 Building and aligning consensus sequences

The starting point for phylogenetic inference is a collection of aligned DNA or protein sequences. The next step in the **TaxMan** pipeline is the production and alignment of consensus sequences for the genes of interest.

5.1 Making consensus sequences

Part of the philosophy of **TaxMan** is the idea that all sequence data that might be relevant to the phylogenetic problem of interest should be stored. A consequence of this approach is the possibility of multiple sequences for a given gene in a given species. Therefore, it is necessary to build a consensus sequence for each gene in each species, before carrying out alignment and further phylogenetic analysis. Obviously, this is only possible because TaxMan only deals with single-copy orthologous genes, where a single sequence can be attributed to each species.

5.1.1 Making gene counts

The method used to construct a consensus sequence for a given gene in a given species depends on the numbers of each different type of sequence. For this reason, the first stage in consensus building is to assemble a list of the numbers of annotated sequences and EST sequences for each gene/species combination. For the purposes of consensus building, **TaxMan** recognises a third category of sequences, genome-derived. These are sequences that were read from records corresponding to a fully sequenced genome (typically a mitochondrial genome or chromosome record).

To make gene counts, select option 3 `(Database step 3 menu)` from the `top level` menu, then select `option 1 (Make gene counts)` from the `Database step 3` menu. **TaxMan** will count up the number of different types of sequences for each gene/species combination.

5.1.2 Building consensus sequences

The actual method of building a consensus sequence for a given gene in a given species depends on the number and types of sequences available. **TaxMan** uses a hierarchical approach with genome-derived sequences preferred over annotated sequences and annotated sequences preferred over EST sequences. If there is a genome-derived sequence available, it is used. If there is no genome-derived sequence, then an annotated sequence is used. If neither of the above are available, EST sequences are used.

If there is more than one EST or annotated sequence available then they are written out into a **FASTA** sequence file and the program **phrap** is used to derive a consensus sequence. This program combines the information from mutiple EST sequences to generate a contig — a contiguous consensus sequence — which can be both longer and higher-quality than the individual sequences. If multiple contigs are produced then the contigs are searched against the **BLAST** databases and the contig with the highest scoring it — usually the longest — is selected as the consensus sequence.

The source of each consensus – genome sequence, annotated sequence, EST sequence or none – is recorded in the record for that particular gene / species combination in the consensus table along with the sequence itself. Finally, **TaxMan** verifies all protein-coding consensus sequence that were constructed from genome-derived or annotated sequences by experimentally translating them in frame 0, using the specified genetic code. This is necessary since the alignment strategy relies on the assumption that such sequences translate fully without internal stop codons; any consensus sequences that do contain stop codons are flagged so that they can be treated appropriately during alignment.

To build consensus sequences, select option 3 `(Database step 3 menu)` from the `top level` menu, then select `option 1 (Make consensus sequences)` from the `Database step 3` menu. **TaxMan** will display the progress as each consensus sequence is built. In cases where a large number of annotated sequences or EST sequences are available, phrap may take a while to build contigs. In these cases TaxMan will display a warning.

5.2 Aligning sequences

For the multiple sequence alignment, **TaxMan** uses **POA** (Partial Order Alignement), an algorithm that uses partial order graphs (section 2.1.2.4). This alignment program has proved to be a good choice for aligning the type of mixed datasets that are typical in **TaxMan**. The reason for this is that it carries out local, rather than global, pairwise alignments. This means that it performs well in situations where some input sequences are very much shorter than others, because end-gaps are not penalised and the short sequences can align to the correct region of the longer sequences

For RNA genes, the alignment process is straightforward. All the consensus sequences for a given gene are written to a FASTA sequence file which is used as the input to POA with the default options.

For protein-coding genes, a more sophisticated method is used. It is desirable to use protein sequences for multiple sequence alignment, especially in cases where the sequences may be highly diverged. Phylogenetic analysis, however, is often carried out at the nucleotide level. TaxMan uses a multi-step alignment program for protein-coding genes. Firstly, all genome sequence– and annotated sequence—derived consensus sequences are translated using the appropriate genetic code, and the protein sequences written out to a FASTA sequence file. This file is then used as the input to POA, with the default options. Then, the aligned protein sequence file is used as a template to back-align the DNA sequences. Finally, the EST-derived consensus sequences are added to this alignment, using the profile alignment option.

To carry out the alignments, select option 4 `(Database step 4 menu)` from the `top level` menu, then select `option 2 (Align Sequences)` from the `Database step 4` menu. **TaxMan** will display the progress as each alignment is carried out. This can be a fairly time-consuming process, depending on the number of sequences and the type of computer on which TaxMan is running.

6 Working with slices

The previous steps, carried out correctly, will result in a database of aligned DNA and protein sequences which can be used to investigate phylogenetic questions within the group of interest. TaxMan produces multiple sequence alignments, in formats suitable for phylogenetic analysis, based on subsets of genes and taxa ('slices'). A slice simply consists of a list of genes and a list of taxa, which exist in the gene table and the species table respectively. By specifying subsets of genes and taxa to with with in this way, the user can produce a wide range of different alignments, containing single or multiple concatenated genes and any number of taxa. Because DNA and protein sequences are stored in their aligned form, producing alignment of slices is very quick, requiring only that the appropriate sequences be retrieved from the database and saved in a suitable format. Because TaxMan assembles each slice when requested, character partitions of various types can be included in the alignment automatically.

6.1 Looking at the distribution of data

Once consensus sequences have been built and aligned, **TaxMan** can produce Comma Separated Value (CSV) files, which can be opened in a spreadsheet application, showing the distribution of sequence data at various taxonomic levels. To do this, select option 5 `(slice menu)` from the `top level` menu, then select `option 3 (Look at the distribution of sequences)` from the `slice` menu. TaxMan will create a number of files:

sources_by_species.csv

This file shows the source of the consensus sequence for each gene in each species. Each row corresponds to a species, and gives the taxid, the binomial name, the class, order and family, then the source for each gene alphabetically. The source can be `n` (none), `g` (genome), `a` (annotated sequence) or `e` (EST sequence).

numbers_by_class.csv
This file shows the number of species in each class that have consensus sequences for each gene. Each row corresponds to a class and gives the name of the class, the number of species belonging to that class in the database, then the number of species in the class that have a consensus sequence for each gene alphabetically.

numbers_by_order.csv
This file shows the number of species in each order that have consensus sequences for each gene. Each row corresponds to a order and gives the name of the order, the number of species belonging to that order in the database, then the number of species in the order that have a consensus sequence for each gene alphabetically.

numbers_by_family.csv
This file shows the number of species in each family that have consensus sequences for each gene. Each row corresponds to a family and gives the name of the family, the number of species belonging to that family in the database, then the number of species in the family that have a consensus sequence for each gene alphabetically.

numbers_by_genus.csv
This file shows the number of species in each genus that have consensus sequences for each gene. Each row corresponds to a genus and gives the name of the genus, the number of species belonging to that genus in the database, then the number of species in the genus that have a consensus sequence for each gene alphabetically

Obviously, depending on the taxonomic level of the group under study, some of the files will be more informative than others.

6.2 Automatically creating slices

TaxMan can create slices automatically. The user can opt to make a default slice for each gene, where the taxa are simply those species for which sequence data is available for the gene. A more sophisticated way to automatically generate slices is to filter taxa based on taxonomic level.

6.2.1 Creating default slices

TaxMan can create a slice for each gene containing only the taxa that have a consensus sequence for that gene. This allows the user to view the complete DNA or protein alignment for a particular gene. To do this, select option 5 `(slice menu)` from the `top level` menu, then select option 1 `(Automatically generate slices)` from the `slice` menu. For each gene in the database, TaxMan will compile a list of taxa where a consensus sequence is available and build a slice. The program will display the slice_id for each of these single-gene slices.

6.2.2 Creating slices based on taxonomic level

A common task in phylogenetics is to infer the relationships between groups at a particular taxonomic level (e.g. all orders in a class, all genera in a family). TaxMan can create a slice that contains a number of species for each higher level taxon that are best-represented for a set of genes. The number of taxa to be included per group, the type of taxon (class, order, etc.) and the set of genes are chosen by the user. This is a useful way to create an alignment that covers the taxonomic diversity of the group under study while containing a reasonable number of species, limiting the computational complexity of phylogenetic analysis.

To do this, select option 5 `(slice menu)` from the `top level` menu, then select option 5 `(Make a slice at a taxonomic level)` from the `slice` menu. You will be prompted to enter a list of genes. Type the canonical names of the genes you want to include in the slice, separated by spaces, or type 'all' to include all genes. You will then be prompted to enter the taxonomic level at which you want to group the species; type one of the options. Finally, enter the number of species you want to include for each group. The program will summarise these specifications (e.g. '3 representatives of each genus will be included') then give the slice_id, the genes that were included and the taxids that were included. The slice_id is a number that is generated automatically. If too many taxids were included (for phylogenetic analysis in reasonable time) you can try again with a higher taxonomic level or fewer included species per group.

6.3 Manually creating slices

If you want to have complete control over the genes and taxa included in a slice, you can manually create one. To do this, select option 5 `(slice menu)` from the `top level` menu, then select option 2 `(Manually create a slice)` from the `slice` menu. You'll be prompted to enter a list of gene names, which should be separated by spaces. The program will then ask if you want to see a list of taxids and species names; you should probably only answer 'yes' if there are only a few species included in the database. Next, enter a list of taxids, separated by spaces. **TaxMan** will save the slice to the database and report the slice_id.

7 Producing an alignment

Once you have defined a slice (a subset of genes and taxa) you can use it to produce an alignment. When you produce an alignment, **TaxMan** reads the list of genes and list of species from the slice table. For each species, it then retrieves the aligned DNA or protein sequence from the database for each gene included in the slice. Finally, it concatenates the aligned sequences for each species and writes a number of files containing the entire alignment. **TaxMan** takes care of removing gap-only columns, inserting the `?` character to represent missing data for any gene where no sequence data is available for a particular species, replacing any terminal gaps with `?`, and setting up character partitions (where supported by the alignment format). Because **TaxMan** uses the pre-aligned sequences, this process is very quick, as no alignment has to be carried out.

7.1 Alignment names and formats

Each time **TaxMan** produces an alignment, it creates a directory with a name based on the slice_id from which the alignment was built. It then creates three files inside the directory, which contain the alignment in different formats – **fasta**, **phylip** and **nexus**. This allows the alignment to be analysed in a range of phylogenetics packages that use these different formats. Nexus format is the only one that allows character partition data.

As an example, if slice_id 21 is used to produce an alignment, the files will be created in a new directory:

slice_id_21_files

For DNA alignments with taxids, the filenames will be:

21.fasta, 21.nexus, 21.phylip

For DNA alignments with species names, the filenames will be:

21.named.fasta, 21.named.nexus, 21.named.phylip

For protein alignments with taxids, the filenames will be:

21.prot.fasta, 21.prot.nexus, 21.prot.phylip

And for protein alignments with species names, the filenames will be:

21.prot.named.fasta, 21.prot.named.nexus, 21.prot.named.phylip

Alignments can be produced with either taxids or species names as labels for the sequences. The choice of label will depend on whether the user intends to use TaxMan to store trees. TaxMan can only store trees where the terminal nodes are labelled with taxids, as that is the only way that species can be associated with the information stored in the species table. Storing trees in TaxMan yields several advantages: stored trees can be output at a later date with various types of annotation; they can be associated with slice_ids and TaxMan can produce a 'pruned' NCBI taxonomy tree for comparison. Alignments labelled with species names can be useful if TaxMan will not be used to store trees, or for visual inspection of the alignment by humans.

7.2 DNA alignments

In addition to character partitions for individual genes, the nexus file for DNA alignments also contains character partitions for each codon position. This makes it easy to apply different models to or exclude bases at different codon positions.

7.2.1 Alignments with taxids

To produce a DNA alignment where the sequences are labelled with taxids, select option 6 `(alignment menu)` from the `top level` menu, then select option 1 `- Produce a DNA alignment from a slice (with taxid codes) -` from the `slice` menu. You'll be prompted to enter a slice_id. TaxMan will then display the genes and species included in the alignment and produce the files. If there are no useful sequences for the chosen species for a particular gene (i.e. that region of the alignment would be all missing data) TaxMan will display a warning and skip that gene.

7.2.2 Alignments with species names

To produce a DNA alignment where the sequences are labelled with species names, select option 6 `(alignment menu)` from the `top level` menu, then select option 2 `- Produce a DNA alignment from a slice (with species names) -` from the `slice` menu. You'll be prompted to enter a slice_id. TaxMan will then display the genes and species included in the alignment and produce the files. If there are no useful sequences for the chosen species for a particular gene (i.e. that region of the alignment would be all missing data) TaxMan will display a warning and skip that gene.

7.3 Protein alignments

7.3.1 Alignments with taxids

To produce a protein alignment where the sequences are labelled with taxids, select option 6 `(alignment menu)` from the `top level` menu, then select option 3 `- Produce a protein alignment from a slice (with taxid codes) -` from the `slice` menu. You'll be prompted to enter a slice_id. TaxMan will then display the genes and species included in the alignment and produce the files. If there are no useful sequences for the chosen species for a particular gene (i.e. that region of the alignment would be all missing data) TaxMan will display a warning and skip that gene.

7.3.2 Alignments with species names

To produce a protein alignment where the sequences are labelled with species names, select option 6 `(alignment menu)` from the `top level` menu, then select option 4 `- Produce a protein alignment from a slice (with species names) -` from the `slice` menu. You'll be prompted to enter a slice_id. TaxMan will then display the genes and species included in the alignment and produce the files. If there are no useful sequences for the chosen species for a particular gene (i.e. that region of the alignment would be all missing data) TaxMan will display a warning and skip that gene.

8 Working with trees

If you use the options above to produce concatenated alignments where the sequences are labelled with taxids, phylogenetic analysis will yield trees where the terminal nodes are also taxids. Such trees can be parsed and stored by TaxMan. The node and tree tables are used to store, along with the structure of the tree, the taxa at the tips, the branch lengths and the support values at each node.

Once a tree has been stored, it can be recalled and written to a tree file along with useful annotation. For instance, tips can be labelled with taxonomic data (such as orders) or sequence statistics. TaxMan can also 'prune' the stored NCBI taxonomy tree to show only the species included in a user tree, complete with annotation of high-level groups. This can be useful when interpreting a user tree.

8.1 Storing trees

Before storing any user trees, the NCBI taxonomy must be stored, in order that taxids can be related to the data held in the species table. Thereafter, user trees can be stored, each of which will be assigned a tree_id that can be used to identify a tree for output.

8.1.1 Storing the NCBI taxonomy

Select option 7 `(tree menu)` from the `top level` menu, then select option 1 `(Store the NCBI taxonomy tree)` from the `tree` menu. TaxMan will read the NCBI taxonomy files and store the information in the database. This may take a few minutes as many records have to be stored – progress will be displayed on the terminal

8.1.2 Storing a newick/nexus tree

Select option 7 `(tree menu)` from the `top level` menu, then select option 2 `(Store a newick tree)` or option 3 `(Store a nexus tree)` from the `tree` menu. Make sure you choose the correct option for the type of tree you want to store. You'll be prompted for a filename, then TaxMan will read and store the file. The tree_id will be displayed after the tree has been successfully stored.

8.2 Outputting trees

To output a tree in **newick** format, you need to know the tree_id. For this reason it's usually a good idea to keep a record of the different slice_ids and tree_ids that you have been working with. By default, when you output a tree **TaxMan** also produces a pruned version of the NCBI tree containing only the species that are in the user tree. The tree files will be stored in a new directory named after the tree id. For example, outputting tree_id `13` would give a directory:

tree_id_13_files

containing the user tree file:

13.newick.nh

and the NCBI tree file:

13.ncbi.nh

8.2.1 Types of annotation

TaxMan can attach various types of annotation to the tips of user trees to aid in interpretation of the tree. The following types of annotation are currently supported:

taxid e.g. `328863`

taxid_scientific name e.g. `328863_Urania_leilia`

scientific name_{order} e.g. `Urania_leilia_{Lepidoptera}`

taxid_common name e.g. `51655_diamondback_moth`

scientific name_common name e.g. `Mamestra_brassicae__cabbage_moth`

8.2.2 Producing a tree

Select option 7 `(tree menu)` from the `top level` menu, then select option 4 `(Output a tree with labels)`from the `tree` menu. When prompted, enter the tree_id. **TaxMan** will display the various annotation options; enter the number of the one you want. It may take a few seconds to output the tree files.

Alphabetic Index

Adding annotated sequences....12
Allpep....14
Allrna....14
Annotated sequences....10
BioPerl....6, 7, 9
BLAST....6, 8, 13
BLAST similarity....13
Blast_database_file....14
Blast_taxa 10
Blastall....8
BLOSUM80....9
Building a BLAST database....13
Canonical name....10
Character partitions....17, 19, 20
Check for common names....13
Comma Separated Value....17
Config....9
Configuration....9, 11
Consensus sequences....11, 15-18
Contig....16
CoreNucleotide....10
Cpan....7
Database....6, 8, 9, 11, 13, 15
Default slices....18
Distribution of data....17
EMBOSS....8
Entrez....10
EST sequences....8, 10, 13, 15, 16
Expressed Sequence Tag....12
Fasta....20
FASTA....14-17
Features....10, 12, 13
Formatdb....8
Gap-only columns....19
Insert gene info....12
Make a blast database....15
Modules....6, 7, 9
Multi-step alignment....17
Nexus....19, 20, 22
Perl....6, 7, 9
Phrap....8, 16
Phylip....19, 20
POA....9
Postgres.log....11
Postgresql....9
PostgreSQL....6, 9
RDBMS....6
RDBMS 9

Records,..........10
Sequence similarity..........12
Slices..........17-19
Synonyms..........10, 12
Taxdump..........11, 15
Taxonomic diversity..........13-15
Taxonomy..........10, 11, 21
Test_script..........9
Tranalign..........8
User trees..........21, 22

Workflow diagram

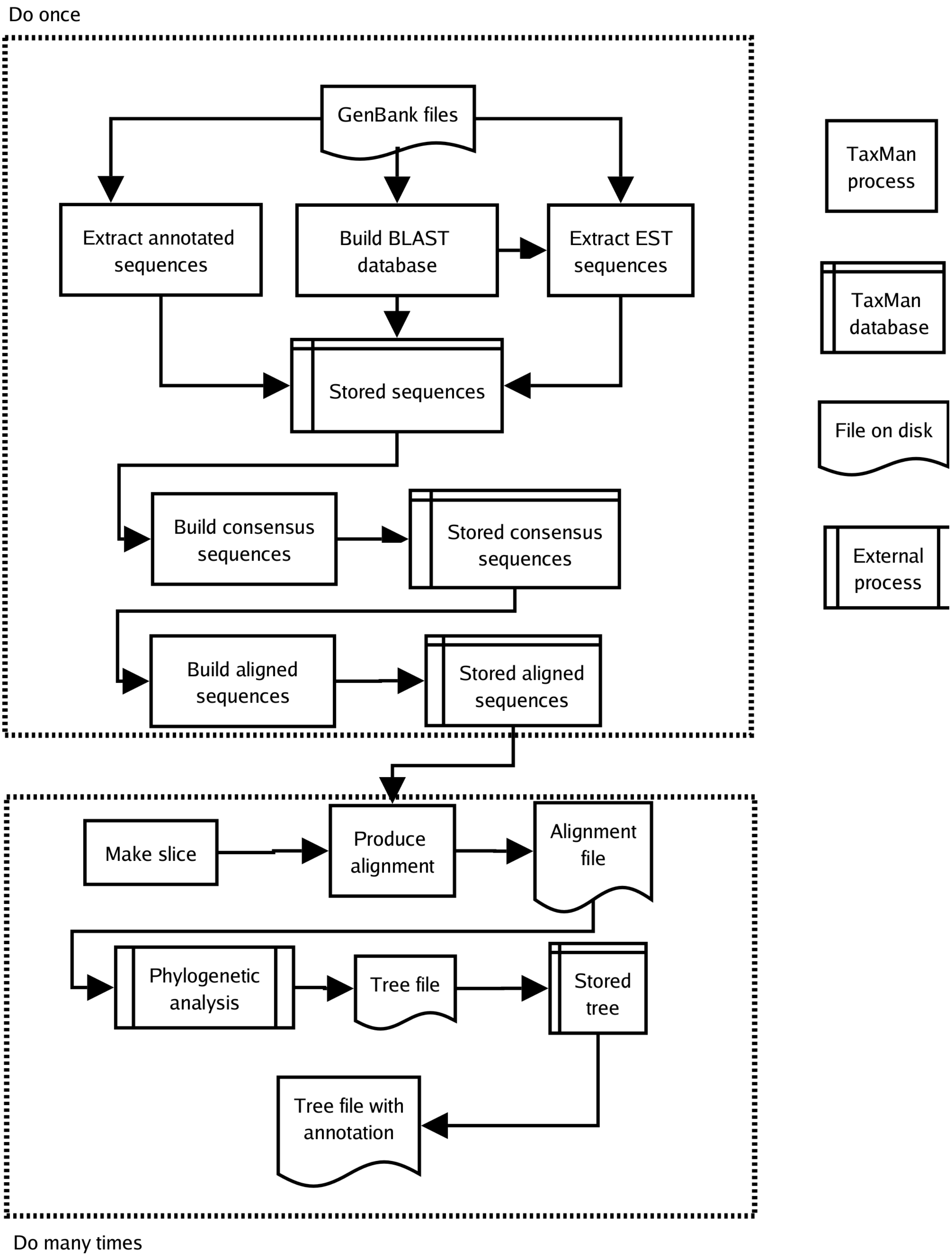

Database diagram

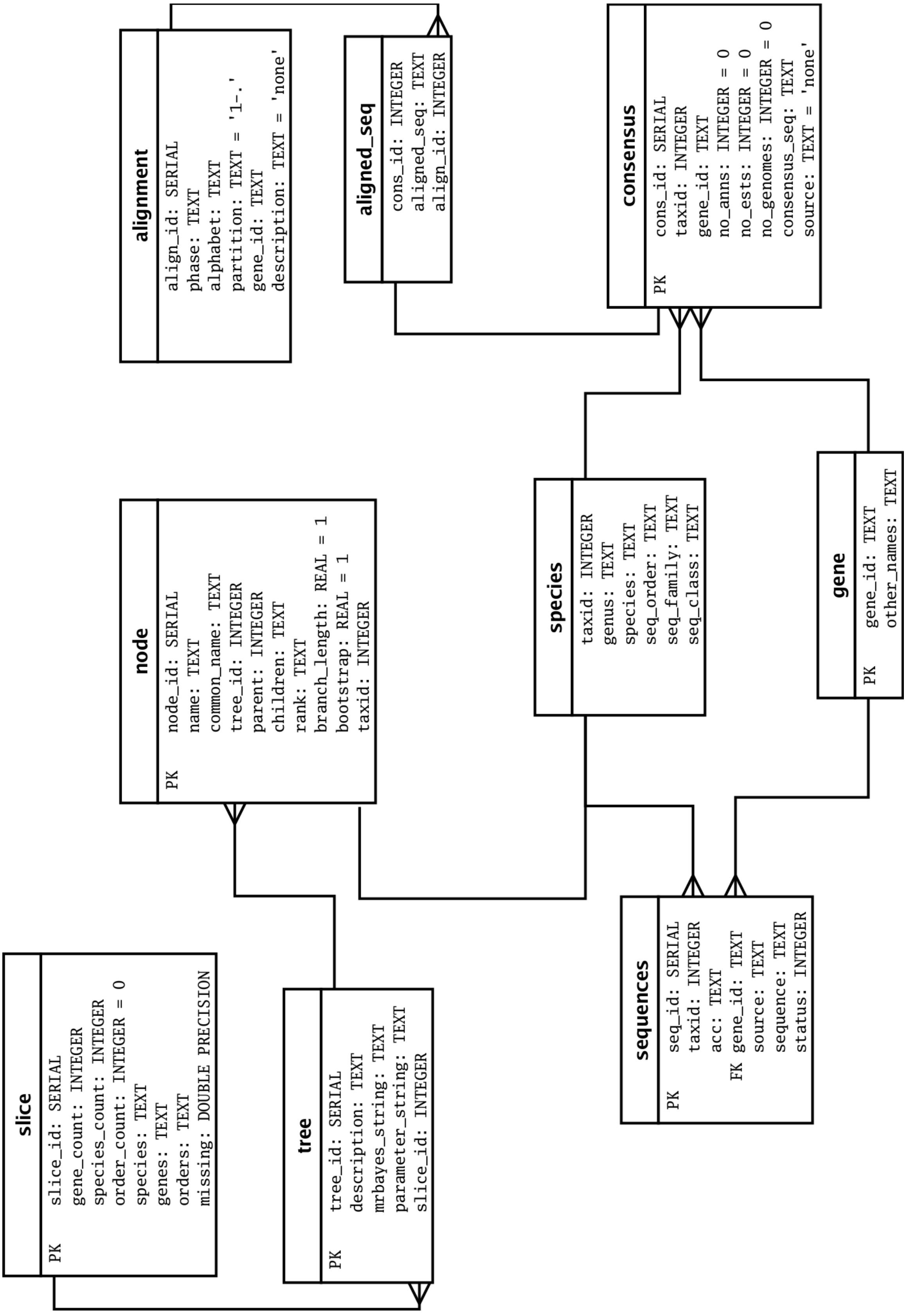

Genetic Codes

1. The Vertebrate Mitochondrial Code
1. The Yeast Mitochondrial Code
2. The Mold, Protozoan, and Coelenterate Mitochondrial Code and the Mycoplasma/Spiroplasma Code
3. The Invertebrate Mitochondrial Code
4. The Ciliate, Dasycladacean and Hexamita Nuclear Code
5. The Echinoderm and Flatworm Mitochondrial Code
6. The Euplotid Nuclear Code
7. The Bacterial and Plant Plastid Code
8. The Alternative Yeast Nuclear Code
9. The Ascidian Mitochondrial Code
10. The Alternative Flatworm Mitochondrial Code
11. Blepharisma Nuclear Code
12. Chlorophycean Mitochondrial Code
13. Trematode Mitochondrial Code
14. Scenedesmus Obliquus Mitochondrial Code
15. Thraustochytrium Mitochondrial Code
